# PARADIGM-SHIFT predicts the function of mutations in multiple cancers using pathway impact analysis

**DOI:** 10.1093/bioinformatics/bts402

**Published:** 2012-09-03

**Authors:** Sam Ng, Eric A. Collisson, Artem Sokolov, Theodore Goldstein, Abel Gonzalez-Perez, Nuria Lopez-Bigas, Christopher Benz, David Haussler, Joshua M. Stuart

**Affiliations:** ^1^Department of Biomolecular Engineering and CBSE, University of California Santa Cruz, 95064 Santa Cruz; ^2^School of Medicine, University of California San Francisco, San Francisco, 94143 CA, USA; ^3^Parc de Recerca Biomedica de Barcelona, E-08003 Barcelona, Spain; ^4^Universitat Pompeu Fabra, E-08002 Barcelona, Spain; ^5^Institució Catalana de Recerca i Estudis Avançats (ICREA), E-08010 Barcelona, Spain; ^6^Buck Institute for Aging, Unit for Christopher Benz is “Cancer Therapeutics Program” 94945 Novato; ^7^Howard Hughes Medical Institute, University of California Santa Cruz, Santa Cruz, 95064 CA, USA

## Abstract

**Motivation:** A current challenge in understanding cancer processes is to pinpoint which mutations influence the onset and progression of disease. Toward this goal, we describe a method called PARADIGM-SHIFT that can predict whether a mutational event is neutral, gain-or loss-of-function in a tumor sample. The method uses a belief-propagation algorithm to infer gene activity from gene expression and copy number data in the context of a set of pathway interactions.

**Results:** The method was found to be both sensitive and specific on a set of positive and negative controls for multiple cancers for which pathway information was available. Application to the Cancer Genome Atlas glioblastoma, ovarian and lung squamous cancer datasets revealed several novel mutations with predicted high impact including several genes mutated at low frequency suggesting the approach will be complementary to current approaches that rely on the prevalence of events to reach statistical significance.

**Availability:** All source code is available at the github repository http:github.org/paradigmshift.

**Contact:** jstuart@soe.ucsc.edu

**Supplementary information:** Supplementary data are available at *Bioinformatics* online.

## 1 INTRODUCTION

A comprehensive cancer survey such as that being generated by the Cancer Genome Atlas (TCGA) program uncovers numerous genomic events in tumors that are a mix of both causal, driving events and neutral, passenger events that accumulate as a result of dysregulated genomic surveillance and cell proliferation with clonal expansion over time. Exome and whole-genome sequencing efforts uncover recurrent mutational events in a few genes and low frequency events in many other genes. Importantly, examples of such low frequency genes are known to be functionally important to disease. For example, although *BRAF*^V600E^ is common in melanoma, it occurs in only 3% of non-small cell lung cancer, but is clearly a driver when present (Dankort *et al.*, 2007).

Although there exist well-described mutations in certain codons of key genes that drive oncogenesis, most somatic mutations in cancer are neutral with respect to overall cell fitness. Many approaches exist to predict the impact of a mutation. However, no existing method incorporates pathway-level information into the assessment of the consequences of a mutation. Investigating the impact of a mutated gene on its pathway neighborhood may provide complementary information to existing approaches.

Current methods often use the frequency of a mutated gene across a cohort, the location of a mutation in the gene, whether the mutations are silent or non-silent, frame-shifting, potentially protein domain altering, found in more evolutionarily conserved regions of the peptide sequence, or cluster together in the protein sequence or structure. Although such methods have shown tremendous success, they have limitations that impact their generality. For example, some methods must be trained from external datasets such as from the COSMIC database, which introduces possible circularity to the analysis and biases the discovery of genes whose mutational impact has already been characterized.

The most popular approach builds gene signatures by training machine-learning classifiers to recognize the presence and absence of mutations from molecular features such as gene expression data (Mooney *et al.*, 2011). These methods can be applied to any number of genomic perturbations including mutations, focal copy number gains or losses, or chromatin methylation events and have the potential of detecting whether mutations in regulatory regions have functional significance. Genes with expression levels that are differentially associated with the presence (compared with the absence) of a mutation are candidates for inclusion in the gene signature using any number of a variety of univariate and multivariate machine-learning and feature selection approaches. One major limitation is that signatures often fail to generalize from one dataset to another.

**Fig. 1.**
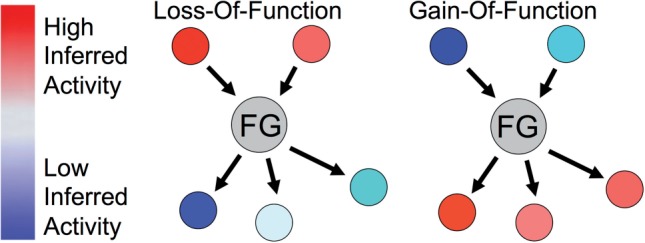
Intuition for predicting the impact of a mutation based on the activity of a FG inferred from its local pathway context. LOF is predicted when FG's downstream targets have activity consistent with a low activity of FG relative to what is expected given its upstream regulators. GOF is predicted when the downstream regulators are consistent with a high activity of FG but the upstream regulators are not

If our pathway knowledge surrounding a particular gene is complete enough and we have enough data to provide information about the activity of neighboring genes, then we can estimate the pathway consequences of a mutation in a tumor sample (Fig. 1). Intuitively, if a mutation influences the function of a focus gene (FG) it may create a particular signature on FG's local pathway neighborhood. In the case of a loss-of-function (LOF) event, the regulatory inputs to the FG would indicate that the gene should be turned on at the transcriptional and/or post-transcriptional levels. For instance, a transcription factor and kinase that regulate different parts of FG's activity may themselves be active. However, when one inspects the activity of genes downstream of FG, one might find evidence that the FG is not active. For example, FG also may be a transcription factor with target genes exhibiting low or undetectable levels of expression. In the gain-of-function (GOF) case, the opposite situation would occur where the downstream targets are consistent with a higher activity of the FG than what would be expected from FG's regulatory inputs.

The method we introduce, PARADIGM-SHIFT, detects a difference in the expected activity of a gene in its downstream neighborhood relative to what is expected given its upstream neighborhood. It makes use of two key pieces of information: (i) the known genetic interactions of a gene; and (ii) the activation or deactivation of these interacting genes to gauge the impact of a mutational event. Since the method uses the data of a sample to contrast the predicted regulatory input from the downstream output of a gene it should provide somewhat orthogonal information for annotating mutations than other approaches.

## 2 METHODS

We derive a PARADIGM SHIFT (*P-Shift*) score based on the intuition of comparing the observed downstream consequences of a gene's activity to what is expected from its regulatory inputs. The *P-Shift* has the form:
(1)
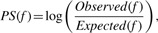

where the ‘expected’ activity of *f* is derived from the upstream regulators and the ‘observed’ activity of *f* is derived from the downstream targets. The caveat of course is that we never get to observe gene *f*'s activity so we must infer it from the activity of its downstream targets, and these activities in a necessarily recursive fashion. The computation can be framed as an inference problem over a set of interdependent variables, some of which are hidden. In the last couple of decades, efficient procedures have been developed to compute the probabilities of a system of random variables connected together in a probabilistic graphical model [see (Friedman, 2004) for a review].

We previously described a factor-graph-based approach for integrating diverse types of omics data with genetic pathways called PARADIGM (Vaske *et al.*, 2010). We briefly summarize the approach here. PARADIGM assesses the activity of a gene in the context of a genetic pathway diagram *ϕ* by drawing inferences from a dataset of observations *D*. The pathway diagram *ϕ* describes connections between hidden gene expression variables, their corresponding observational data, and any regulatory inputs and outputs. Variables are connected to each other by *factors*, which encode probabilistic dependencies constraining mutually connected variables. The dataset can include multiple different types of measurements for a patient sample such as gene expression and genomic copy number variation. Before supplying the data to PARADIGM, each dataset type is first transformed into rank-ratios. This is done by ranking all values across all samples from smallest to largest and then each rank *r* is transformed into the range [0,1] by the formula (*r* – 1)/(*N* *G – 1) where *N* is the number of samples and *G* is the number of genes measured. All data and hidden states are represented in PARADIGM as ternary random variables in which the value *x^a^* encodes more active in the tumor than normal, *x^i^* more inactive in the tumor and *x*^0^ equal levels. Briefly, PARADIGM then uses a belief-propagation algorithm on a factor graph derived from *ϕ* to compute *inferred pathway levels* (IPLs) for each gene, complex, protein family and cellular process by combining gene expression, copy number and genetic interactions. The IPL for a gene is a signed log-posterior odds (LPOs) of the state of the gene given the observed data. Positive IPLs reflect how much more likely the gene is active in the tumor, whereas negative IPLs reflect the negative log probability of how likely the gene is inactive in the tumor relative to normal.

Our contribution here is the development of a method that can predict the impact of a mutation in a tumor sample using two calls to the PARADIGM algorithm for each mutated gene. We first describe the computation of a score that reflects the predicted neutrality, loss- or GOF of a mutational event. The method provides a prediction for each gene and each sample in the cohort and thereby provides a sample- or patient-specific assessment of the functional impact of a mutation. The computation assumes a local pathway context for the gene is given. However, the second section describes how a gene's pathway context is selected. Finally, we describe how we then compute cohort-wide measures of significance to determine if a gene is more often involved in loss- or GOF events.

### 2.1 Computation of the *P-Shift* score

**Fig. 2.**
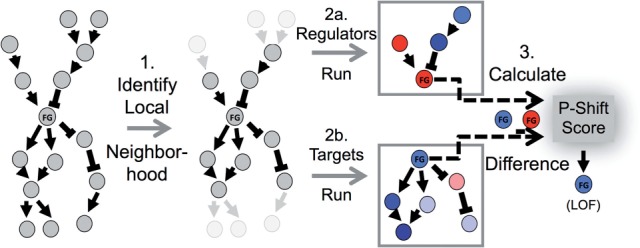
Overview of the PARADIGM-SHIFT method. Inference is centered on a FG for which mutations have been detected in one or more samples. First, a local neighborhood around FG is isolated from the full pathway. PARADIGM is run in two modes using only the local neighborhood. A ‘Regulators Run’ infers the activity of FG using only its upstream regulators in the local neighborhood; a ‘Targets Run’ infers FG's activity using only downstream targets. Finally, the difference between these two runs is calculated and used as the *P-Shift* Score. In this case, LOF is predicted because the downstream targets indicate lower activity than the upstream regulators of FG

The core of our approach estimates a *P-Shift* score for each tumor sample and for each FG using two runs of the original PARADIGM algorithm (Fig. 2). We refer to these two runs as the Regulators-only and the Targets-only runs (R-run and T-run for short). In the R-run, a neighborhood of upstream regulators is left connected to FG but all downstream targets are disconnected. The inferences derived from the R-run reflect the expected level of FG given the state of its regulators in a particular sample. In the T-run, FG is left connected to a neighborhood of its downstream targets while upstream regulators are disconnected. The *P-Shift* score then computes the difference between the inferred activities of FG determined in the T-run from those determined in the R-run.

To estimate pathway-neighborhood dependent inferences on FG *f*′ s activity, we restrict our view of the data to subsets of features in *ϕ* and supply these as arguments to *D*. Let *R* ⊂ *ϕ* be the set of regulators of *f* and *T ⊂ ϕ* be the set of targets. The selection of *T* and *R* is described in the next sub-section and assumed given here. Let *D*(*R*),* D*(*T*) and *D*(*f*) refer to the data observed for the regulators, targets and the FG *f*, respectively. The interactions are restricted to the subset of features in the FG's neighborhood and denoted *ϕ*(*T*) to represent the pathway features and interactions involving only the targets to each other and to *f* itself. Similarly, *ϕ*(*R*) represents the same for the upstream regulators. With these definitions in hand we write the *P-Shift* score as the following log ratio of two constituent likelihood-ratios:
(2)
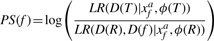

where *LR*(*Y*|*x^a^, Z*) is defined as *P*(*Y*|*x^a^*,*Z*)/*P*(*Y*|*x*^¬^*^a^*,*Z*), the likelihood ratio computed over one possible alternative value for *X, x^a^*, compared with the probability of the other two possible values—less active in tumor *x^i^* and similarly active in tumor *x*^0^—the combined event is written for short. Note that only the expected term in the denominator contains the entry *D*(*f*), which represents the actual data for the FG of interest. This reflects the assumption that the data on the FG provides evidence for the *cis*-regulatory state of the gene and so is included among the regulators of *f*. Note that if data on the direct activity of *f* were instead available, such as phosphorylation status or enzymatic activity, then that data could be considered for inclusion into the numerator term for the observed targets. The quantity in Equation (2) reflects the degree to which the observed data for the targets is consistent with high activity of the FG relative to the observed data for the regulators and the gene in question.

Further expansion of the *P-Shift* score reveals the method by which it can be computed using the original PARADIGM algorithm. Application of Bayes Rule gives:
(3)
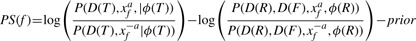

where *prior* is the log-prior-odds and has the same form as the first two terms in the equation except that all entries involving *D* are dropped. Another application of Bayes Rule would show that the first two joint probability ratio terms are equivalent to the LPO that the gene is active given either the state of the downstream targets (left-hand term) or the upstream regulators (right-hand term). The advantage of writing the joint probabilities in this form shows explicitly those terms of the form *P*(*D, x* | *ϕ*) that are each efficiently computed with a message-passing belief propagation procedure on the underlying factor graph encoded by *ϕ.* The message-passing procedure sums out all of the hidden variables present in *ϕ*—the states of complexes, cellular processes and the activities of all other genes other than *f*. The computation implements an iterative form of the Expectation–Maximization procedure that sequentially updates all variables by forming a running average until either a convergence tolerance of 10^−9^ is reached or 10 000 maximum iterations are exceeded. The code is freely available through the libDAI C++ open source library (Mooij, 2010). In the R-run version of PARADIGM, the LPO shown in Equation (3) and its corresponding log-prior odds are computed in two separate full factor graph convergence runs. Likewise, the T-run involves two separate EM runs to compute its two terms in Equation (3). Thus, in total, the computation time involved to compute the *P-Shift* requires four EM convergence runs, but each task is run on a reduced pathway representation involving only the neighborhood of the FG. Thus, the computation time to calculate a *P-Shift* for an entire dataset requires 2*k* PARADIGM runs where *k* is the number of mutated genes in the cohort, usually on the order of dozens with a minimum frequency of exonic mutations.

In practice, we use the IPLs from PARADIGM for the computation of the *P-Shift*. Specifically, we set PS(f)=IPL*_T_* (*f*)-IPL_R_(*f*), where IPL_T|R_(*f*) is the IPL derived from the T- or R-run. The IPL is a signed LPO that always puts the highest probability state for *f* in the numerator. If the inactive state is in the numerator the IPL gives the negation of the LPO. This quantity is similar to Equation (3) except that the highest probability states determined in each run are contrasted. In the case where the active form of the gene is the most probable in each case the two formulas are equivalent. Finally, we found that a transformation of the *P-Shift* score to a Z-score provided better overall results. Each gene's local neighborhood could have a certain bias to lean toward either positive or negative scores. To account for this, we constructed 100 random samples for each gene by shuffling data tuples around *ϕ*. This effectively associates random data with each gene's neighborhood. *P-Shifts* were calculated for each of these 100 samples and each *P-Shift* was then normalized by subtracting the mean and dividing by the standard deviation determined from this simulation. We henceforth refer to these *P-Shift* Z-scores simply as the *P-Shifts.*

### 2.2 Selection of the local pathway context

Rather than use the full set of interactions in the SuperPathway, the set of regulators R and targets T are selected from a local neighborhood *L* around each gene to make computation feasible. All interactions between the selected features are included in the neighborhood. If a protein is present in both the upstream and downstream neighborhoods due to feedback circuitry it is excluded from both the R- and T-runs. To build the neighborhood, we traverse the graph and include any pathway features (proteins, families, complexes and processes) if there exists a path from the neighbor to FG that includes no more than *k* intervening proteins. We expect *L* to provide a good approximation of the full network, as genes further away will exert a diminishing influence on the inference of the FG. To test this intuition, we repeated all analyses on the positive controls with *k*= 0, 1, 2, 3 and *L* set to the full network. As expected, we found that including genes further away from the FG provided little influence on the *P-Shift* score with *k >* 1 showing little difference (data not shown). To enrich the neighborhood for genes with informative data we used a simple variance filter in which neighbors in *L* having less than a 0.10 standard deviation in the rank-ratio-transformed expression data were excluded.

To further test the method and shed light on comparisons with gene expression-signature-based approaches, we also implemented a supervised neighborhood selection step. Neighbors in *L* are selected if their expression values have a minimum covariance with the presence and absence of FG mutations in a training set. Five-fold cross-validation is performed in which a Student's *t*-statistic is calculated for each gene in *L* using 80% of the samples and those with at least a *t*-statistic of 1.0 are retained. This amounts to applying a relaxed Fisher criterion as a feature selection step for classification. The *P-Shift* scores for FG are then calculated over the held-out 20% of the samples in the test set. The process is repeated for each fold. The same cross-validation partitioning was used to train a Support-Vector Machine (SVM) to represent a competing gene expression signature-based approach for comparison (Supplementary Fig. S4A).

### 2.3 Datasets and pathway sources

We downloaded gene expression, copy number and exome-capture mutation data for patient tumor samples from the TCGA data coordinating center for 185 glioblastoma multiforme (GBM) samples on 7/28/11, 354 ovarian serous denocarcinoma (OVCA) samples on 7/28/11, 219 colorectal carcinoma (CRC) samples on 7/28/11, 184 lung squamous carcinoma (LUSC) samples on 10/26/11 and 525 breast carcinoma (BRCA) samples on 9/24/11. Datasets for each tissue-specific tumor type were used separately as the dataset *D* for inferring mutation *P-Shifts*. We formed a comprehensive cellular pathway diagram for *ϕ* by merging together several pathway sources into a superimposed pathway henceforth referred to as the ‘SuperPathway’ (Heiser *et al.*, 2011).

## 3 RESULTS

**Fig. 3.**
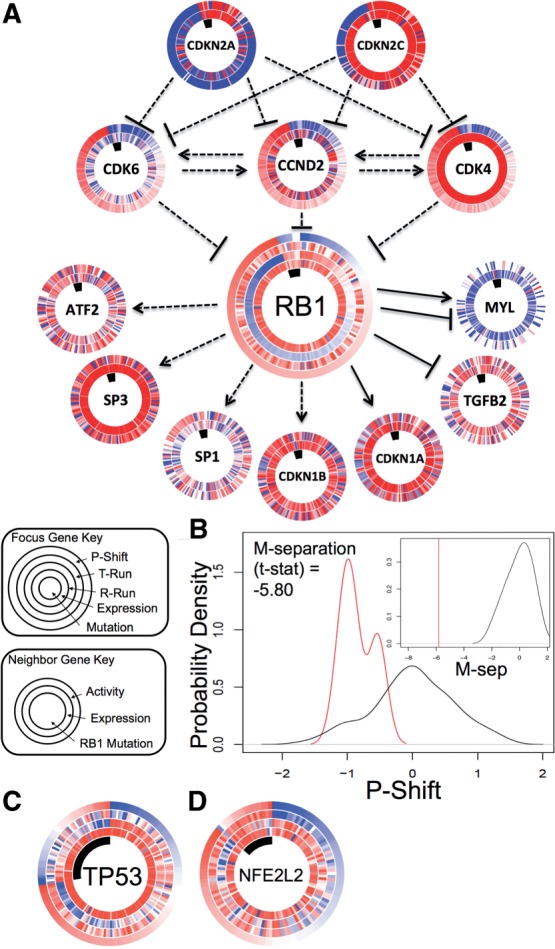
PARADIGM-Shift results on three example genes. (**A**) CircleMap display shows RB1 results on GBM. From innermost to outermost ring: mutation status, expression level, activity derived from the Regulators-Run, activity from the Targets-Run and the *P-Shift* score; spokes are samples ordered first by mutation status of RB1 and then by increasing *P-Shift* score in the clockwise direction. Neighbors of RB1 have their inferred activities from either the T-run for targets or the R-run for regulators. (**B**) *P-Shift* scores (*X*-axis) calculated for samples harboring mutations in RB1 (red histogram) plotted alongside *P-Shift* scores calculated for samples lacking a reported RB1 mutation (black histogram). *Y*-axis shows probability density proportional to the fraction of samples with a given score. The distributions are significantly different as reflected by a Student's *t*-test (*t* = −5.78). Inset, m-separation score summarizing the difference in the mutant versus non-mutant *P-Shift*s (red line) is significantly lower than a random background simulation in which random neighboring genes were associated with RB1 (black histogram). *X*-axis, m-seps; *Y*-axis, probability density as in part (C). (**C**) CircleMap for TP53 in GBM. Same coloring as in part (A). (**D**) CircleMap of NFE2L2 in LUSC. Same coloring as in part (A)

We applied PARADIGM-SHIFT to a set of three well-characterized genes including *RB1*, *TP53* and *NFE2L2*. The retinoblastoma (RB1) gene is a well-known tumor suppressor gene and plays a crucial role in the control of the G1→ S transition of the cell cycle. We applied our method to predict the functional consequences of mutations to *RB1* in the GBM cohort. Neighborhood selection identified 6 proteins in the upstream neighborhood of RB1 and 10 downstream targets. The expression data, mutation status of RB1, and the inferences from the T- and R-runs for this neighborhood are shown as integrated CircleMaps in Figure 3A. Of these, 6 out of 9 samples received negative *P-Shift*s consistent with RB1's characterized role as a tumor suppressor. At a fixed FPR (*α* = 0.10), 4 out of 9 RB1 mutants are detected with a one-sided test.

We asked whether the distribution of *P-Shift* scores were significant by computing a *mutant separation t-*statistic (m-sep) comparing the *P-Shifts* associated with the mutated samples to the *P-Shifts* associated to those samples without reported RB1 mutations (Fig. 3B). It is important to note that the unsupervised version of the method makes no use of the mutation calls in any way when deriving the score. Although most of the samples received negative *P-Shifts*, a number of RB1 mutations had scores closer to the mean of the non-mutated cases. These events may reflect neutral passenger mutations that happen to land in the RB1 gene, or might reflect heterozygous as opposed to homozygous events.

Although the m-seps indicate that the distributions of the *P-Shift* scores are appreciably lower for the mutants compared with the non-mutants, we performed a permutation analysis to assess whether the observed m-seps are significant using a non-parametric approach. We formed random neighborhoods for RB1 by assigning data tuples from random genes to the regulators and targets of RB1. Using 1000 different sets of randomly assigned neighbors the entire procedure was repeated and the difference between the mutant and non-mutant distributions were computed. This test indeed revealed that the lower *P-Shifts* observed for the mutant RB1 samples were significantly lower than the non-mutants relative to those differences seen in random controls (Fig. 3B, inset).

*TP53* is the most commonly mutated gene in cancer. It is a tumor suppressor and in most cases dominant negative mutations or deletion of the gene resulting from loss of heterozygosity (LOH) or double somatic events are observed as early and frequent events in cancers spanning many tissue types. We compared our algorithm's ability with predict the functional impact of *TP53* mutations in OVCA and GBM available through the TCGA consortium. In GBM, nearly a third (48) of the samples have a mutation in *TP53*. Of these, 19 had negative *P-Shifts*, 9 had positive *P-Shifts* and the rest had near-neutral as determined by permutation analysis. In OVCA, the majority (67%) of the samples had a reported *TP53* mutation. Importantly, it is believed that nearly 100% of the samples harbor such a mutation even though less than 100% were detected (Consortium, 2011). Figure 3C shows the CircleMap view of TP53's data, inferred activities from T- and R-runs, and its *P-Shift* scores. It is clear that the *P-Shifts* in the left-hand side, corresponding to the mutated cases, are enriched for lower scores, consistent with a LOF prediction for this gene. The difference in *P-Shifts* for mutated versus non-mutated were again found to be significantly left-shifted (Supplementary Fig. S1A, m-sep = −10.9).

To gauge the utility of the method in predicting GOF mutations on a known proto-oncogene, we applied our method to mutations in NFE2L2 in lung squamous cell carcinoma. NFE2L2 is a transcription factor that directs response to stress and oxidative damage in cells. Activating mutations in specific lysine residues stabilize the protein by preventing its degradation via the KEAP1/CUL3 ubiquitin ligase complex. In the TCGA lung squamous dataset, 17 samples harbored mutation in NFE2L2. Of these, 6 had positive *P-Shifts*, 1 had negative *P-Shifts* and the rest were in the neutral range as determined by permutation analysis as shown in the CircleMap in Figure 3D. At a fixed FPR (*α*= 0.10), 3 out of 17 NFE2L2 mutants are detected with a one-sided test and 6 of 17 with a two-sided. Random permutation analysis confirmed that the positive *P-Shifts* seen for the mutated cases relative to the non-mutated cases were significant. Thus, our method was able to predict a positive increase in activity of this gene relative to its regulatory inputs consistent with the known constitutively activating influence of these mutations.

**Fig. 4.**
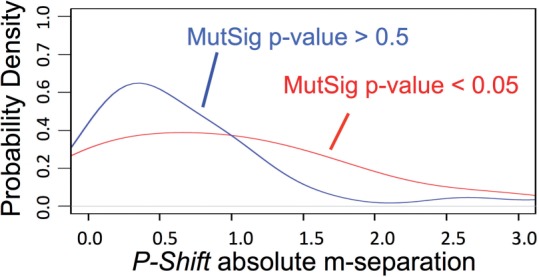
*P-Shift* and MutSig are correlated on the GBM cohort. *P-Shift* m-seps (*X*-axis). The m-seps for those genes with significant MutSig *P*-values were plotted (red distribution) separately from genes with insignificant MutSig *P*-values (blue distribution)

To gauge the general agreement with predicting functional impacts for mutated genes that are considered to be driver rather than neutral passenger events, we compared our approach with MutSig (Getz *et al.*, 2007). MutSig considers the frequency of the mutation, the location of the mutation in the gene, and several other features to calculate significance score relative to an estimated sample-specific background mutation rate. We collected all MutSig scores for those genes that had representation in the SuperPathway and that had at least three mutations in GBM. We used the absolute value of the average of the *P-Shifts* and dividing them into two groups according to whether they were associated with significant or insignificant MutSig scores (Fig. 4). The results show a clear enrichment for higher absolute *P-Shifts* (either indicative of GOF or LOF) for those genes with significant MutSig scores compared to those with insignificant scores.

Next, to determine a rough estimate for the specificity of our approach, we collected six of the genes that received insignificant MutSig scores on which to perform the aforementioned permutation analysis. We plotted the *P-Shifts* from the permuted samples and found that in each of the six cases, the calculated m-seps fell well within the range seen in the permuted controls (Supplementary Fig. S2). We find that these mutations in genes with low MutSig scores are associated with *P-Shifts* that do not discriminate between mutant and non-mutant samples, consistent with the assumption that many of these mutations represent passenger events. Thus, our pathway-based method shows a degree of confirmation to a purely sequence-based analysis of mutational events.

**Fig. 5.**
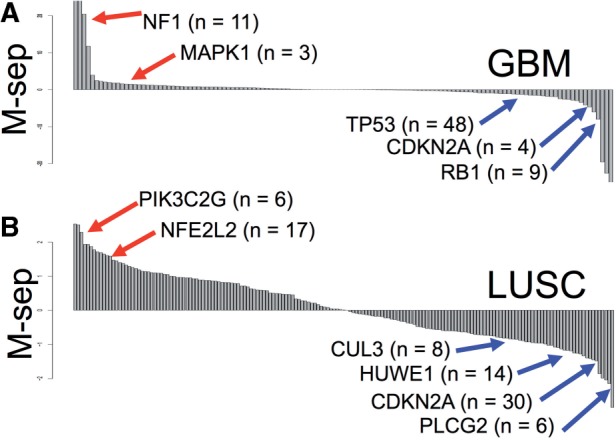
PARADIGM-Shift predicts several low frequency mutations as impactful in multiple cancers and is very uncorrelated to sequence-based assessment methods. (**A–B**) *P-Shift* results on several cancer cohorts. All axes plot the m-separation between mutant and non-mutant samples (*Y*-axis) for each gene (*X*-axis). Genes are ordered by their associated m-seps. (A) Results for GBM, and (B) lung squamous cell (LUSC) are shown

As discussed above, most methods to assess significance of mutations in a given gene rely heavily on the prevalence of mutations across a clinical cohort with shared characteristics (e.g. early stage colon cancer). However, some rare events are of paramount importance to the patients in whom they occur, such as in the Ras-MAPK pathway in GBM. To determine novel impactful events, we applied PARADIGM-SHIFT to all of the mutated genes in GBM, OVCA and LUSC. For each cohort, we calculated all *P-Shift*s, computed an average *P-Shift* for each gene, and plotted these averages as ‘waterfall’ plots to highlight examples of predicted gains- and losses-of-function (Fig. 5A–C).

Our analysis identified probable GOF mutations in MAPK1 based on only 3 out of 171 samples sequenced in GBM (Fig. 5A). All three lie in the protein kinase domain and two are predicted to change the kinase function due to their occurrence in highly conserved residues in the kinase, suggesting kinase inhibitors targeting the ERK proteins in select cases may be effective. CDKN2A is a well-known tumor suppressor whose loss, primarily through homozygous copy number deletion, is an early driver of oncogenesis. Thus it is consistent that our method predicts LOF for this important tumor suppressor in both GBM and LUSC (Fig. 5B) for those cases in which the gene is present but mutated. Counter to expectation, our method assigns a positive score to NF1, which is a well-known tumor suppressor gene whose loss in GBM also defines a distinct transcriptional subtype {PMID: 20129251}. Thus, pathway utilization likely differs substantially not only between mutant and wt cases, but also between cancer subtypes, possibly making analysis of such marker events complex.

In addition to NFE2L2 discussed earlier, analysis of the LUSC cohort (Fig. 5C) also reveals potentially therapeutically important targets in select cases. PIK3C2G, for example, may act as a driver in a handful of patients and could in theory be targeted by AKT pathway inhibitors or rapalogs. The low negative value of HUWE1, a less well-studied E3 ligase, suggests this enzyme might play a role analogous to that of CUL3 in degrading NFE2L2 in some cases.

PARADIGM-SHIFT applied to the ovarian dataset also gave informative insights into this tumor type. Nearly all samples harbor TP53 mutations (*n* = 179). The *P-Shifts* were mostly negative for TP53 consistent with the expected LOF of this tumor suppressor. In addition, our analysis may clarify potentially important directional information about pathway alterations. For example, the EPH receptor family is known to participate in bidirectional signaling (Aoto and Chen, 2007). The high absolute differences in the shift scores for this diverse family in the ovarian cohort may reflect functionally opposing roles of these bidirectional receptors in oncogenesis.

Finally, we asked whether the PARADIGM-SHIFT method provides complementary information to current techniques by comparing it to both a gene-expression signature-based and several popular sequence-based approaches. The absolute *P-Shift* score was used as an indicator for the presence of a mutation. To further inform the comparison of the method to a gene-signature-based approach a supervised version of the *P-Shift* was implemented based on a *t-test* and referred to as *P-Shift-t* (see Section 2). For the gene expression-signature-based approach a linear kernel SVM was used. Two SVM models were trained—one that used the entire set of genes in the dataset and a second that used only the genes in the local neighborhood *L*. Supplementary Fig. S4A shows the results of predicting the presence/absence of the mutation with the *P*-Shift and *P-Shift-t* methods compared with the SVM for the three positive control genes. The *t*-test supervision does seem to either help or provide comparable performance (e.g. TP53) compared with the original unsupervised *P-Shift*. Interestingly, the SVM-based approaches limited to the *P-Shift* neighbors outperformed the full set of features in all these cases. In addition, except for RB1, both variants of the *P-Shift* give lower performance than the SVM-based approach.

We compared *P-Shift* with four different approaches including SIFT (Kumar *et al.*, 2009), PolyPhen2 (Adzhubei *et al.*, 2010) and MutationAssessor (Reva *et al.*, 2007). CONDEL (Gonzalez-Perez and Lopez-Bigas, 2011) was also included as it produces an integrated call by combining the above three methods. We calculated the Pearson's correlation between each of the methods and between each of the methods to the absolute *P-Shift* and displayed the results as a heatmap (Supplementary Fig. S4B). Not surprisingly, due to the heavy sequence-based nature of the previous methods, they all have higher correlations among themselves than they do to *P-Shift*. Still, a moderate level of correlation was observed between *P-Shift* and sequence-based methods, achieving the highest level (0.30) with the integrated CONDEL method. Thus, our method may provide novel viewpoints on mutations that can be used in conjunction with sequence-based methods.

## 4 DISCUSSION

Our approach uses different information compared with protein-sequence-based approaches. It enables probing into infrequent events and can be used to detect the impact of non-coding mutations. In addition, it may be useful for detecting those cases that harbor passenger mutations where the mutation is either neutral or the cell has compensated somehow to keep the surrounding pathway intact. Finally, since our approach couples single gene mutation events with broader pathway activation signatures, it could be used to place genes with unknown/little known function and provocative mutations, into new pathways, as suggested by the case of HUWE1.

The method we describe has several limitations. Although absolute *P-Shifts* show a good overall correlation with MutSig, several of the genes seem to have predictions on average in the opposite direction than expected (e.g. NF1 in GBM). Complex regulatory logic surrounding the gene may show a discrepancy but the direction of the discrepancy may not always be clear. It will take further investigation into these cases to determine if a reliable direction can be inferred from the sign of the *P-Shift* score.

The selection of the upstream and downstream pathways are key steps for accurate prediction of events in individual samples. Because of the combinatorial complexity of the selection problem, research is needed to determine a neighborhood that gives maximum performance at identifying the presence and absence of mutations. Our results do indicate that even mild supervision of the neighborhood selection step gives slightly better performance. In addition, an SVM gene expression signature-based approach provides higher accuracy in predicting the presence/absence of mutations. Since the SVM cannot be used to gauge the loss- versus GOF of a gene, using the *P-Shift* together with the SVM might improve the discrimination between mutant and non-mutant samples as indicated in Supplementary Fig. S4A but it will be interesting to see if the directionality of the shift is also improved. In this investigation, it would be informative to perturb neighborhood members, connections, parameters and neighborhood selection policies to measure how the performance varies to errors in the pathways.

Finally, the method can only be applied to genes with sufficient representation in the curated set of pathway interactions. Although current pathway databases have a biased coverage of cancer-related genes, many of the genes with low-frequency mutations are still among those with little pathway information. It is critical to expand pathways beyond the curated set to encompass such orphan genes into the analysis of mutation consequences. Indeed, current efforts are underway to expand pathway databases by including high-throughput functional genomics results (Wu *et al.*, 2010). These efforts should greatly improve the breadth of genes to which pathway-based mutation impact approaches can be applied.
